# Changes in Bacterial Resistance Patterns of Pediatric Urinary Tract Infections and Rationale for Empirical Antibiotic Therapy

**DOI:** 10.4274/balkanmedj.2015.1809

**Published:** 2017-09-29

**Authors:** İbrahim Gökçe, Neslihan Çiçek, Serçin Güven, Ülger Altuntaş, Neşe Bıyıklı, Nurdan Yıldız, Harika Alpay

**Affiliations:** 1 Department of Pediatrics, Division of Pediatric Nephrology, Marmara University School of Medicine, İstanbul, Turkey

**Keywords:** Urinary tract infection, antibiotic resistance, children

## Abstract

**Background:**

The causative agent spectrum and resistance patterns of urinary tract infections in children are affected by many factors.

**Aims:**

To demonstrate antibiotic resistance in urinary tract infections and changing ratio in antibiotic resistance by years.

**Study Design:**

Retrospective cross-sectional study.

**Methods:**

We analysed antibiotic resistance patterns of isolated Gram (-) bacteria during the years 2011-2014 (study period 2) in children with urinary tract infections. We compared these findings with data collected in the same centre in 2001-2003 (study period 1.

**Results:**

Four hundred and sixty-five uncomplicated community-acquired Gram (-) urinary tract infections were analysed from 2001-2003 and 400 from 2011-2014. Sixty-one percent of patients were female (1.5 girls : 1 boy). The mean age of children included in the study was 3 years and 9 months. Escherichia coli was the predominant bacteria isolated during both periods of the study (60% in study period 1 and 73% in study period 2). Bacteria other than E. coli demonstrated a higher level of resistance to all of the antimicrobials except trimethoprim-sulfamethoxazole than E. coli bacteria during the years 2011-2014. In our study, we found increasing resistance trends of urinary pathogens for cefixime (from 1% to 15%, p<0.05), amikacin (from 0% to 4%, p<0.05) and ciprofloxacin (from 0% to 3%, p<0.05) between the two periods. Urinary pathogens showed a decreasing trend for nitrofurantoin (from 17% to 7%, p=0.0001). No significant trends were detected for ampicillin (from 69% to 71%), amoxicillin-clavulanate (from 44% to 43%), cefazolin (from 39% to 32%), trimethoprim-sulfamethoxazole (from 32% to 31%), cefuroxime (from 21% to 18%) and ceftriaxone (from 10% to 14%) between the two periods (p>0.05).

**Conclusion:**

In childhood urinary tract infections, antibiotic resistance should be evaluated periodically and empiric antimicrobial therapy should be decided according to antibiotic sensitivity results.

Urinary tract infections (UTIs) are an important public health problem in children. If treatment is delayed or inadequate, recurrent UTIs in particular can lead to permanent damage including renal parenchymal scarring, poor kidney function, high blood pressure and even chronic kidney disease (1).

UTIs are treated empirically until urine culture results are available. Due to unnecessary and inappropriate use of antibiotics and the failure of empirical treatment in community-acquired UTIs, antibiotic resistance is increasing among uropathogens, especially among Gram-negatives, worldwide (2).

Physicians should know the antibiotic susceptibility patterns of uropathogens to determine appropriate empirical therapy. When prescribing empirical therapy for community-acquired UTIs, we should be aware of resistance patterns in our community. Because antibiotic resistance patterns may change over time in the same geographical region, continued monitoring is required. Therefore, in the present study, we studied the changing susceptibility patterns of commonly used antibiotics in community UTIs and, in particular, in community-acquired Gram (-) UTIs in children over a 10-year period.

## MATERIALS AND METHODS

### Patients

In this retrospective study, the study population consisted of children aged from 2 months to 16 years living on the Asian side of İstanbul, who were admitted or consulted with culture-proven Gram (-) UTIs to the Paediatric Nephrology Clinic between January 2011 and December 2014 (study period 2). We compared these findings with data collected in the same centre from January 2001 to December 2003 (study period 1) which have already been published (3). Samples were collected from children with a suspected UTI. Patients who had history of previous hospitalization (<30 days) and genitourinary abnormalities were excluded.

### Sampling, identification of bacterial species and susceptibility testing

To obtain urine samples, a midstream clean-catch method was used in children who were toilet trained and transurethral catheterization was performed in non-toilet-trained children. A UTI was defined if bacterial growth was >105 colony-forming units (CFU)/mL in midstream urine culture or >104 CFU/mL in urine culture obtained by the transurethral route in addition to the clinical features. Gram (+) pathogens were excluded from the study. Antibiotic susceptibility patterns of isolated urinary pathogens were examined with an automatized identification technique (VITEK, Biomerieux, Marcy-l’Etoile, France). The resistance pattern of cultured Gram (-) bacteria to antimicrobials (ampicillin, amoxicillin-clavulanate, trimethoprim-sulfamethoxazole, cefazolin, cefuroxime, ceftriaxone, cefixime (first, second and third generation cephalosporins), amikacin (an aminoglycoside) and ciprofloxacin (a fluoroquinoline) was identified. Written informed consent was obtained from at least one parent and ethical approval was obtained from the Marmara University School of Medicine ethics committee (B.30.2.MAR.0.01.02/AEK/120187481).

### Statistical analysis

Statistical analysis was performed using The Statistical Package for the Social Sciences 16.0 (SPSS Inc., Chicago, Illinois, USA). The frequency of microorganisms and their resistance to the tested antibiotics were compared for two time periods, namely, 2001-2003 and 2011-2014 using the chi-square test. The accepted level of significance was 0.05.

## RESULTS

Four hundred and sixty-five uncomplicated community-acquired Gram (-) UTIs were analysed in study period 1 (2001-2003) and 400 in study period 2 (2011-2014). Sixty-one percent of patients were female (1.5 girls : 1 boy). In study period 1, 59% of patients were female (1.42 girls : 1 boy) whereas in study period 2, 64% of patients were female (1.75 girls : 1 boy). The mean age of children included in the study was 3 years and 9 months (between 2 months and 16 years) (in study period 1, mean age 3.1 years and in study period 2, mean age 4.5 years).

*Escherichia coli* was the predominant bacterium isolated during both periods of the study (60% in study period 1 and 73% in study period 2) followed by *Klebsiella* spp. and other Gram (-) bacilli including *Proteus* spp., *Pseudomonas spp*. and *Enterobacter* spp. (Table 1). Table 2 shows the antibiotic resistance rates of the bacteria other than *E. coli*. The bacteria other than *E. coli* demonstrated a higher level of resistance to all of the antimicrobials except trimethoprim-sulfamethoxazole than *E. coli* bacteria. Table 3 shows the changes and trends in antibiotic susceptibility rates of cultured microorganisms by period. In our study, we found increasing resistance trends of urinary pathogens for cefixime (from 1% to 15%, p<0.05), amikacin (from 0% to 4%, p<0.05) and ciprofloxacin (from 0% to 3%, p<0.05). Urinary pathogens showed a decreasing trend for nitrofurantoin (from 17% to 7%, p=0.0001). No significant trends were detected for ampicillin (from 69% to 71%), amoxicillin-clavulanate (from 44% to 43%), cefazolin (from 39% to 32%), trimethoprim-sulfamethoxazole (from 32% to 31%), cefuroxime (from 21% to 18%) and ceftriaxone (from 10% to 14%) between the two periods (p>0.05).

## DISCUSSION

We should know the antibiotic resistance rates of a living area to determine appropriate empiric therapy for UTIs. Antibiotic resistance, especially in underdeveloped and developed countries, may change and usually increases over time in the same geographical region due to the use of unsuitable antibiotics (3,4,5,6,7). The increasing resistance to antimicrobials like amoxicillin-clavulanate and trimethoprim-sulfamethoxazole in children is an important problem for paediatricians.

Amoxicillin-clavulanate and trimethoprim-sulfamethoxazole are the favourite empiric treatment options for uncomplicated UTIs in Turkey (3). The most frequent drugs prescribed for parenteral treatment are third generation cephalosporins and aminoglycosides. Trimethoprim-sulfamethoxazole, amoxicillin-clavulanate and nitrofurantoin are commonly chosen drugs for prophylactic treatment.

The most striking result of our study is that almost all the bacteria tested showed high resistance to commonly used oral antibiotics. Approximately 50% of the microorganisms showed resistance to amoxicillin-clavulanate and 30% to trimethoprim-sulfamethoxazole and first generation cephalosporins. According to some authors we shouldn’t use drugs empirically if their resistance rate exceeds the threshold value of 20% (2). Such levels would render trimethoprim-sulfamethoxazole, first generation cephalosporins and amoxicillin-clavulanate inadequate for empirical treatment. Eighteen percent resistance to cefuroxime and 15% resistance to cefixime lead us to recommend these drugs as alternative drugs for empiric treatment.

The use of oral cefixime was suggested by Hoberman et al. (8) in 1999 instead of intravenous antimicrobials for febrile UTIs in children. We also found that third generation cephalosporins were effective for most of the microorganisms in our study population (Table 3). We also do not prefer hospitalization versus outpatient treatment, but it is sometimes necessary for UTI patients who are under the age of 3 months, patients who have a risk of urosepsis, patients with a urinary tract malformation or patients with vomiting and dehydration. We should prefer narrow-spectrum antimicrobial therapy if the clinical presentation of the patient is convenient for outpatient treatment. Another important point is the rising resistance to third generation cephalosporins, especially to cefixime, in patients with UTIs. This is the most rapidly increasing resistance rate identified in this study. It is well known that unnecessary use of antimicrobial drugs for UTIs and other types of infections causes high resistance rates to antibiotics (9). To minimize the development of antibiotic resistance, prescriptions of third generation cephalosporins should be avoided as much as possible and may be reserved to the treatment of pyelonephritis.

The drugs most frequently prescribed for parenteral treatment are third generation cephalosporins and aminoglycosides. In this study, we found acceptable antibiotic resistance rates of 14% for ceftriaxone and 4% for aminoglycosides (Table 3). These results suggest that third generation cephalosporins and aminoglycosides are convenient for sufficient parenteral therapy for UTI in our population.

It is well known that widespread use of cephalosporins is associated with the emergence and spread of extended spectrum beta-lactamases (10). In light of our findings, in patients with UTIs, we suggest starting empirical parenteral treatment with an aminopenicillin and aminoglycoside combination instead of third generation cephalosporins. This regimen is cheap, working also against enterococci, and is not likely to promote resistance.

Prophylactic drugs used for UTIs in children are nitrofurantoin, amoxicillin, amoxicillin-clavulanate and trimethoprim-sulfamethoxazole. The results of our study showed that amoxicillin-clavulanate resistance was 43%, trimethoprim-sulfamethoxazole resistance was 31%, nitrofurantoin resistance was 7% and cefuroxime resistance was 18%. Nitrofurantoin is an oral drug which showed good activity against microorganisms in the present study. According to our findings we recommend the use of nitrofurantoin for prophylaxis. We do not prefer nitrofurantoin in children with febrile UTIs, because although it has sufficient urinary concentrations, it fails to reach therapeutic concentrations in the bloodstream (11). In special circumstances, another alternative treatment for prophylaxis may be cefuroxime with 18% resistance rates.

As a result, trimethoprim-sulfamethoxazole is not the drug of choice for empirical treatment, whereas cefuroxime and cefixime are suitable drugs for the initial treatment of community-acquired UTIs. For parenteral treatment, third generation cephalosporins and aminoglycosides are still effective. The most convenient drug for prophylaxis seems to be nitrofurantoin according to our study.

Bacterial resistance to commonly used antibiotics is increasing. Therefore, it is extremely important to achieve a strict antibiotic prescription policy. Antimicrobial resistance rates change over time, highlighting the need for continued monitoring of the susceptibility patterns of uropathogens. Empirical treatment and prophylaxis of the patients with UTIs should be decided according to local resistance patterns.

## Figures and Tables

**Table 1:**
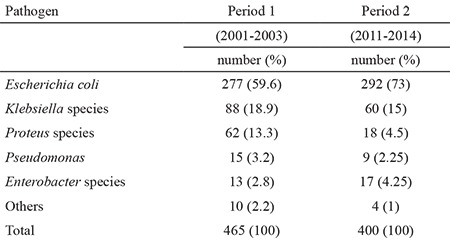
Isolated Gram (-) pathogens during both periods of the study

**Table 2:**
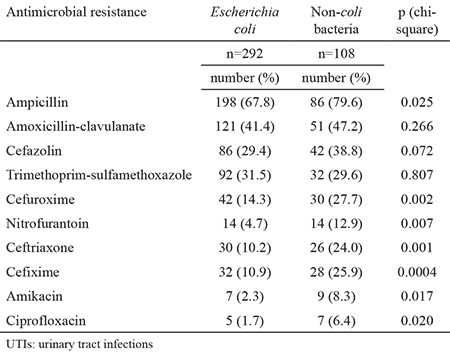
Antibiotic resistance pattern of bacteria (Escherichia coli & non-coli bacteria) isolated from children with acquired UTIs* in İstanbul, 2011-2014

**Table 3:**
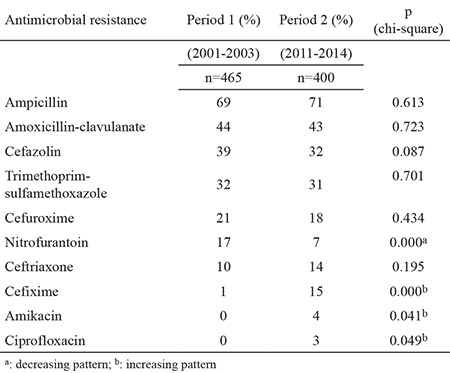
Changes in antibiotic resistance patterns of isolated Gram (-) pathogens by years
